# Effects of sex and soil water chemistry on leaf morphology and physiology of *Myrica gale* var. *tomentosa*

**DOI:** 10.1371/journal.pone.0275024

**Published:** 2022-09-22

**Authors:** Inoue Mizuki, Yoshiharu Sango, Kiyoshi Ishida, Yuko T. Hanba, Masaaki Chiwa, Yoshitoshi Uehara, Atsushi Kume

**Affiliations:** 1 Department of Biosciences, College of Humanities and Sciences, Nihon University, Setagaya−ku, Tokyo, Japan; 2 Department of Biology, Faculty of Agriculture and Life Science, Hirosaki University, Hirosaki, Aomori, Japan; 3 Department of Applied Biology, Kyoto Institute of Technology, Sakyo–ku, Kyoto, Japan; 4 Kyushu University Forest, Kyushu University, Sasaguri, Fukuoka, Japan; 5 Research Institute for Human and Nature, Kamigamo, Kita−ku, Kyoto, Japan; 6 Faculty of Agriculture, Kyushu University, Nishi-ku, Fukuoka, Japan; Universite du Quebec a Chicoutimi, CANADA

## Abstract

Plants respond to environmental stressors, such as an oligotrophic environments, by altering the morphological and physiological functions of their leaves. Sex affects these functions because of the asymmetric cost of reproduction in dioecious plants. We compared the leaf mass per leaf area (LMA), ratio of intercellular air space in leaf mesophyll tissue (mesophyll porosity), palisade thickness, and carbon isotope ratio (δ^13^C) of leaves of the dioecious shrub *Myrica gale* based on sex and gradients of soil water chemistry across habitats in the field. The PCA showed that the first three principal components accounted for 84.5% of the variation. PC1 to PC3 were associated with the origin of soil water, nitrogen status of habitats, and sea–salt contributions, respectively. LMA varied from 5.22 to 7.13 μg/cm^2^, and it was positively related to PC2 and negatively related to PC3, but not to PC1 or sex, suggesting that LMA was low under poor nitrogen conditions and varied with salinity. Mesophyll porosity values were over 50% for all habitats. Mesophyll porosity was positively affected by PC3 and smaller in females than in males. This suggests that *M*. *gale* exhibits differences in mesophyll anatomy according to sex. Palisade thickness ranged from 0.466 to 0.559 mm/mm. The leaves of females had thinner palisade layers per mesophyll layer than those of males; however, the habitat did not affect the thickness of the palisade layer per mesophyll layer. The δ^13^C values of leaves varied from −32.14 to −30.51 ‰. We found that δ^13^C values were positively related to PC2 but not to PC1, PC3, and sex. Under poor nitrogen conditions, the δ^13^C of *M*. *gale* leaves decreased, suggesting that nutrient deficiency would decrease more under the long-term averaged ratio of photosynthesis than stomatal conductance, leading to low water use efficiency.

## Introduction

Plants adapt to different environments by altering leaf characteristics. Stressors such as shade, drought, and/or oligotrophic environment affect leaf morphological and physiological functions, such as photosynthetic traits and water potential [1–4]. Leaf morphological characteristics are one of the determinants of photosynthesis [5]. Therefore, differences in the morphology and physiology of leaves along environmental gradients can change the photosynthetic ability of plants, leading to differences in plant growth and reproduction [6–8].

Dioecious plants, that is, plants that produce male/female flowers on different individuals, are found in approximately 6% of angiosperms [9]. In many dioecious species, spatial sex segregation across habitats has been reported [10, 11], suggesting that environmental gradients have different effects between sexes on survival, growth, and reproduction. The higher cost of reproduction in females than in males in dioecious plants imposes different resource demands on the sexes. These differences lead to differences in leaf morphology and physiology between sexes. The morphology and physiology of the leaves of a dioecious plant, *Hippophae rhamnoide* L., differs between sexes along an altitudinal gradient [12]. Water availability, such as drought stress, strongly affects the morphology and physiology of leaves more in females than in males [8, 12]. Furthermore, these morphological and physiological differences between sexes were sometimes observed before reproductive maturity, suggesting that these differences were not necessarily caused by the latest trade-off in resources between growth and reproduction of individuals but by genetic factors [13, 14].

*Myrica gale* var. *tomentosa* L. (Myricaceae) is a dioecious wetland clonal shrub distributed from northeast Asia to northwest North America. It coexists with symbiotic N–fixing bacteria of the genus Frankia [15]. Thus, it obtains nitrogen from the air and tolerates nitrogen deficiency in its habitat. However, when the potassium concentration in the soil water of habitats decreases, sex ratios at the flowering ramet level becomes biased toward males in *M*. *gale* [16]. The sex ratio has also been biased towards males in habitats where female leaves showed low phosphorus concentrations [17]. An oligotrophic environment causes differences in reproduction between sexes because fruiting requires high amounts of potassium and phosphorus. Furthermore, directly and indirectly, deficiency of potassium and phosphorus might lead to differences between sexes in reproduction through differences between sexes in photosynthetic ability in oligotrophic environments.

We expected that the habitat environment and sex would affect the morphology and physiology of leaves in the dioecious shrub *M*. *gale*. We focused on leaf mass per area (LMA), ratio of intercellular air space in leaf mesophyll tissue (mesophyll porosity), and palisade thickness, which are related to light capture availability, and carbon isotope ratio, which is related to long–term water use efficiency and as a physiological trait of leaves. We hypothesized that females would have a made smaller LMA, smaller mesophyll porosity, thinner palisade thickness, and lower carbon isotope ratio than those of males because females would invest more resource in reproduction, causing poor availability of resources for growth, especially in oligotrophic environments. To estimate whether there were oligotrophic environments, we investigated soil water chemistry.

We also focused on the female-absent population. Populations of *M*. *gale* sometimes showed an absences of females [16, 18, 19]. These populations were more isolated and smaller than those of other *M*.*gale* populations (smaller than 2,000 m^2^). These female-absent populations are locally endangered and require continuous monitoring [16]. We considered that excessive sex ratio distortion of *M*. *gale* in oligotrophic environments occurred in these populations. Thus, we hypothesized that the values of various leaf traits in the female-absent population would show outliers.

## Materials and methods

### Study species and sites

*Myrica gale* var *tomentosa* L. is a dioecious wetland shrub that spreads clonally via root suckers [16]. The sex of ramets in this species can be identified when flowers bloom. The sexual phenotypes of ramets rarely changed over a 3-year observation period (the change was less than 0.3%; Mizuki et al., unpublished data). They flower immediately after snow melts and are pollinated by wind. The roots of *M*. *gale* contain symbiotic N-fixing bacteria of the genus *Frankia* [15].

**Fig 1:**
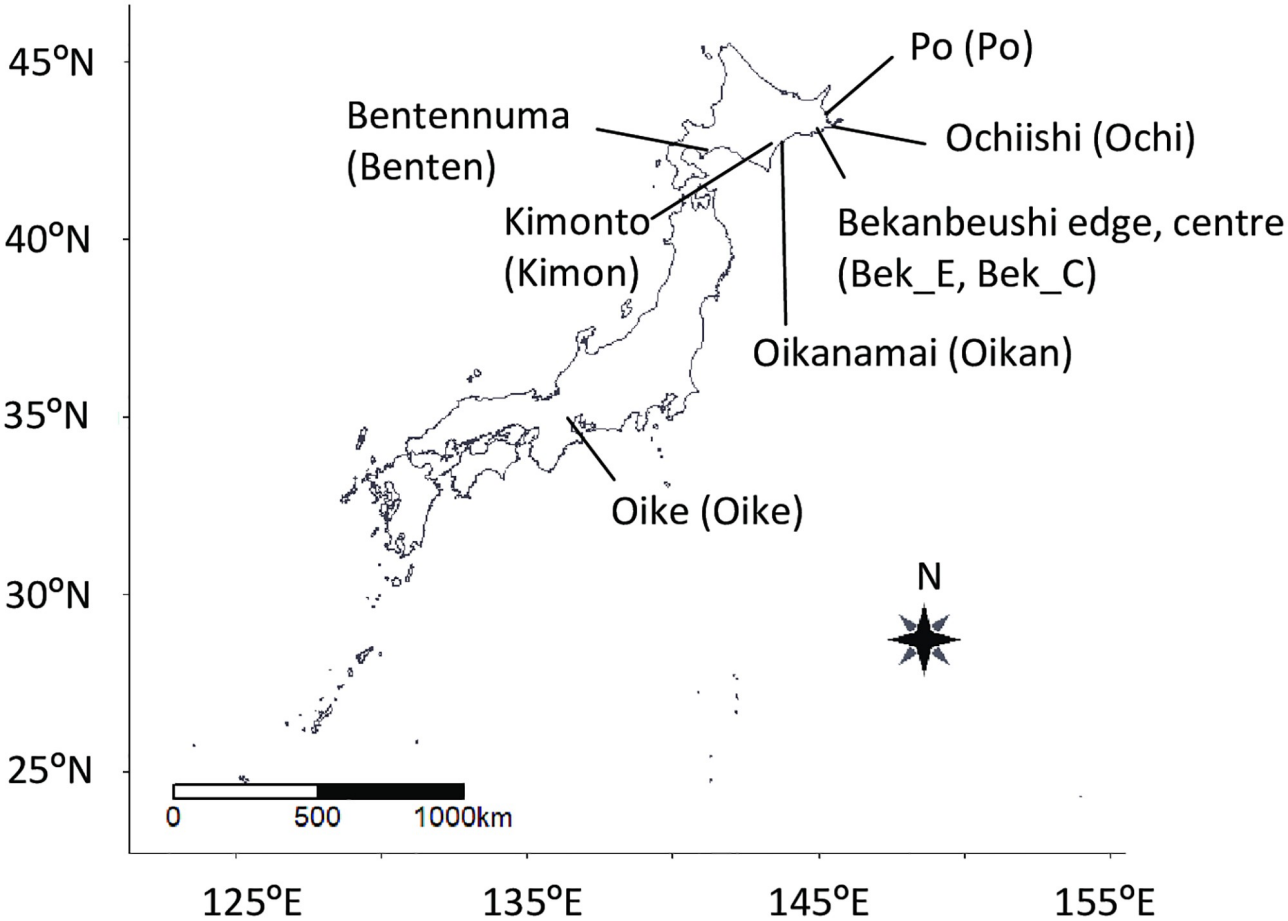
Study sites of *Myrica gale* populations. Abbreviations of the sites are shown in parentheses. Maps were created using Natural Earth. Free vector and raster map data @ naturalearthdata.com.

We surveyed eight populations of *M*. *gale*: populations at Oike (annual precipitation, AP = 1771 mm), populations at the edge and center of Bekanbeushi Moor (AP = 1065 mm), and populations at Bentennuma (AP = 1012 mm), Kimonto (AP = 1065 mm), Oikanamai (AP = 1110 mm), Ochiishi (AP = 944 mm), and Po (AP = 1128 mm) (Fig 1). Although the center of Bekanbeushi Moor was only about 1 km from the edge, *M*. *gale* at the center of the Bekanbeushi Moor was never soaked in water (high moors, bog) and those at the edge were always soaked in water (fen). Therefore, the soil water chemistry differs from each other [12]. In addition, the sex ratios of the sites were similar, but the flowering ratio at the edge was nine times higher than that at the center [16]. Thus we selected two populations at the site. Oike, which is a female-absent population, is the smallest habitats (smaller than 1,600 m^2^) among the study sites (larger than 7,500 m^2^). Further information about the study sites is provided by [12, 17]. We studied these sites with the permission of the Japanese Forestry Agency and Education Boards.

### Soil water chemistry

To investigate soil water chemistry of habitats, soil water was collected using five suction soil water samplers (DIK−8392, DAIKI), which consisted of a ceramic porous cup, lead pipe, and syringe. They buried in the soil to extract the soil water at a depth of 30 cm at each site. Sampling points were set at least 20 m apart from each other, and we attempted to choose these points to cover the area range of leaf sampling. However, we sampled three points in Oike because its habitat was less than 1,600 m^2^ and was severely restricted for vegetation conservation. All samples were rapidly stored in 50 ml polypropylene bottles, and the filtered samples were stored in the dark at 4°C until chemical analysis. Water samples were filtered through cleaned 0.7 μm glass fiber filters (Whatman, GF/F). Soil water samples were collected on July 30, 2019, at Oike, August 22 at Bentennuma, August 23 at Oikanamai, August 24 at Kimonto and the edge on Bekanbeushi Moor, August 25 at the center of Bekanbeushi Moor, Ochiishi, and August 26 at Po.

The pH of each sample (10 ml) was measured using a glass electrode (Horiba, F54S) and EC with a conductivity meter (Toa, CM−60V). For dissolved total nitrogen (DTN) analysis, filtered samples (5 ml) were digested in an alkaline solution of potassium peroxydisulfate (NaOH−K_2_S_2_O_8_). For dissolved total phosphorus (DTP) analysis, samples (15ml) were digested in potassium peroxydisulfate (K_2_S_2_O_8_). The DTN and DTP contents were measured using ultraviolet absorptiometry (Shimadzu UV mini−1240 spectrophotometer) and molybdenum blue (ascorbic acid) absorptiometry (Shimadzu UV mini−1240), respectively. The dissolved SiO_2_ concentrations in the filtered samples (5 ml) were determined using the molybdenum yellow method (see [20] for details). Filtered samples (1 ml) were passed through a 0.45 μm membrane filter (GL Science, Chromatodisc, 25A) to quantify the major ions (Cl^−^, NO_3_^−^, SO_4_^2−^, Na^+^, NH_4_^+^, K^+^, Mg^2+^, and Ca^2+^). The major ions were analyzed using an ion chromatograph (Dionex, DX−120). Dissolved organic nitrogen (DON) was calculated by subtracting NO_3_^−^ and NH_4_^+^ from DTN.

### Morphology and physiology of leaves

Three male and three female ramets were selected from each habitat. However, there were no females in Oike, we chose only three male ramets. We selected from the 2nd to 6th leaves counting from the shoot apex.

To clarify the anatomical characteristics of the leaves, 5 mm × 2 mm leaf sections were obtained from the lamina per sex per site (3 individuals × 2 sexes × 7 populations and 3 individuals × 1 sex × 1 Oike population), avoiding the midrib. They were infiltrated with 2.5% glutaric aldehyde buffer and then fixed in 2% osmium tetroxide buffer. Thereafter, the sections were dehydrated in an ethanol series and embedded in Spurr resin (ERL−4206, NishinEM, Tokyo) according to standard procedures. Thin sections (700 nm) were obtained using an ultramicrotome (ULTRACUT N, Reichert−Nissei, Tokyo) for light microscopy using a microscope (BX51–33, OLYMPUS, Tokyo, Japan) digitally recorded with a CCD camera (VB–7010, KEYENCE, Osaka, Japan). The digitized images were analyzed using ImageJ software [21]. We measured the thickness of the mesophyll, palisade, and spongy layers in four different fields of the two sections (four fields × two individuals × two sexes × seven populations and four fields × two individuals × one sex × Oike population). To measure mesophyll porosity, we used three sections per sex per site (three individuals × two sexes × seven populations and three individuals × one sex × Oike population), and the width of the section images was 100 μm.

To estimate leaf mass per leaf area (LMA), we obtained three sections of leaves per sex per site (three individuals × two sexes × seven populations), measured leaf area using a scanner (Canoscan 9950F, Canon, Tokyo) and ImageJ, and weighed the dry mass after drying the leaves at 60°C for 48 h in an oven (MOV−112, SANYO, Osaka).

Three leaf disks (0.5 cm^2^) were cut from the leaves per sex per site (three individuals × two sexes × seven populations and three individuals × one sex × Oike population) and the dry mass was weighed. The stable isotope signatures and C contents of the leaf disks were determined using the UC Davis Stable Isotope Facility. We determined the leaf carbon isotope composition (δ^13^C) as follows:

$$\delta^{13}\text{C}\;\left(‰\right)=\left\{\left[{\left({}^{13}\text{C}\;{/}^{12}\text{C}\right)}_{\text{sample}}-\;{\left({}^{13}\text{C}\;{/}^{12}\text{C}\right)}_{\text{standard}}\right]\;/\;{\left({}^{13}\text{C}\;{/}^{12}\text{C}\right)}_{\text{standard}}\right\}\times 10^{3}$$

### Statistical analysis

The R software program [22] was used for all statistical analyses. Principal component analysis (PCA) was performed to assess the variance among the different soil water chemistry of the habitats.

As the following analyses were used to clarify the effect of sex, we removed the data from the female-absent population, Oike, from the following analyses. To evaluate whether sex and habitat affected the LMA, we used a Gaussian generalized linear model (GLMs) to analyze the effect of principal component scores of PC1 to PC3 and sex on the mass of leaves across seven populations (Bentennuma, Oikanamai, Kimonto, edge, and center on Bekanbeushi Moor, Ochiishi, and Po). The models were offset by the leaf area. To evaluate whether sex and habitat affected the thickness of the palisade layer, we estimated the effect of the principal component scores of PC1 to PC3 and sex on the thickness of the palisade layer using a Gaussian generalized linear mixed model (GLMMs) with the sections as a random effect. The models were offset by the thickness of the mesophyll layer. We used log-linked Gaussian GLMs to analyze the effect of the principal component scores of PC1 to PC3 and sex on mesophyll porosity across the seven populations. We used GLMs to analyze the effects of principal component scores of PC1 to PC3 on the δ^13^C values of leaves across seven populations. These models were simplified using the Akaike information criterion (AIC).

## Results

### Habitat environmental conditions

**Fig 2:**
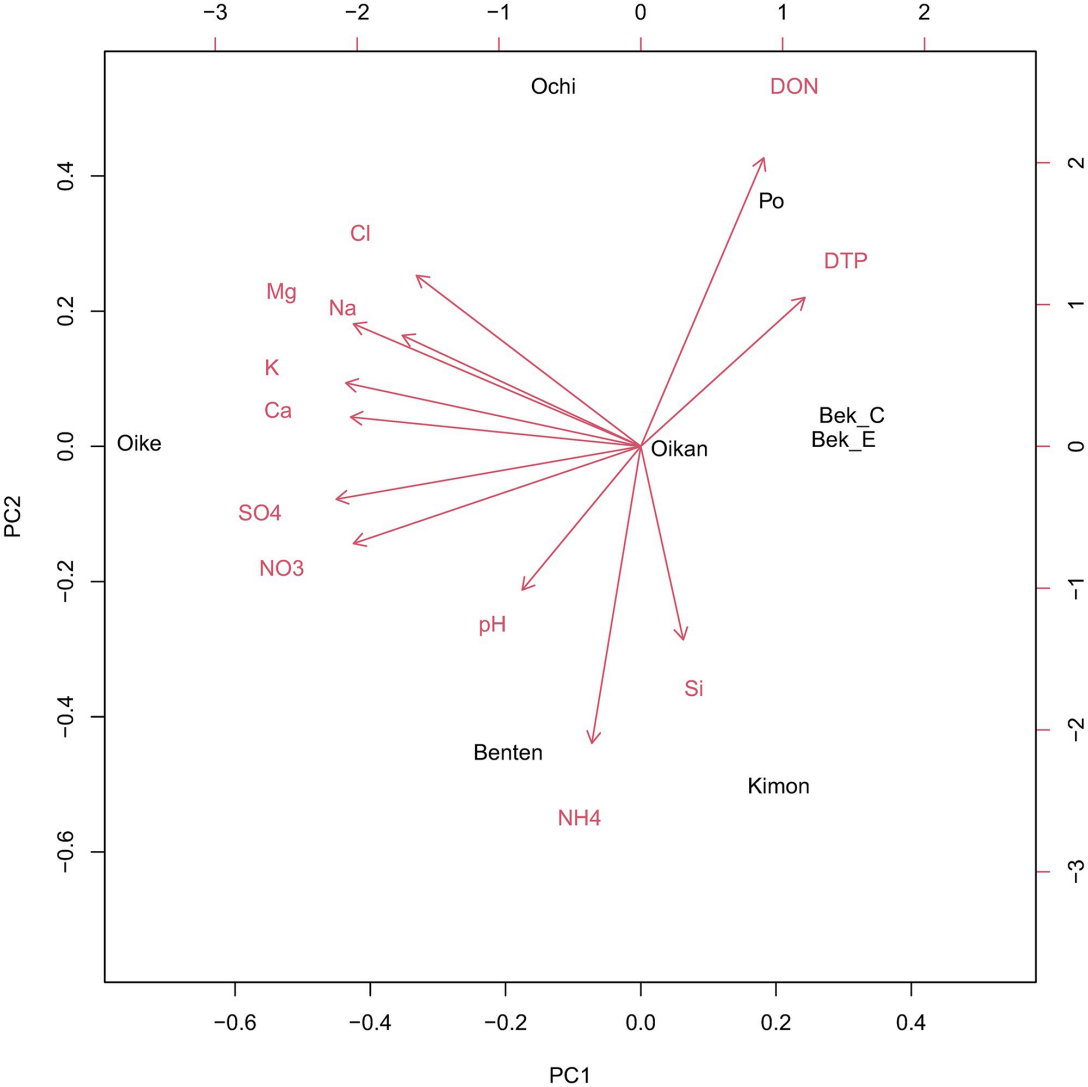
Principal component analysis (PCA) plot of eight *Myrica gale* var. *tomentosa* populations. Red arrows indicate the parameters used as variables of soil water chemistry. Black characters indicate the abbreviation of populations in Table 1; Oike: Oike, Po: Po, Ochi: Ochiishi, Bek_C: Bekanbeushi_Center, Bek_E: Bekanabeushi_Edge, Oikan: Oikanamai, Kimon: Kimonto, Benten: Bentennnuma.

We examined the chemistry of the soil water (Table 1). K^+^ ranged from 20.99 to 76.92 μmol/L. DON varied among 75.25 and 178.62 μmg/L. DTP varied among 14.19 and 98.33 μg/L. Three PCs explained 84% of the variance of the normalized dataset of the soil water chemistry (Table 2). The first component (PC1) contributed 46% variance and revealed negative associations with NO_3_^−^, SO_4_^2−^, K^+^, Mg^2+^, and Ca^2+^ (Fig 2). PC2 contributed 30% variance and revealed positive associations with DTN and DON and negative associations with NH_4_^+^ and Si. PC3 contributed 8% variance and revealed strong associations with Na^+^ and Cl^−^ and negative associations with Ca^2+^. The populations are shown in Fig 2.

**Table 1:** Summary of chemical analyses of soil water in eight habitats of *Myrica gale*.

Habitat	Oike	Po	Ochiishi	Bekanbeushi		Oikanamai	Kimonto	Bentennuma
				Center	Edge			
Ab.	Oike	Po	Ochi	Bek_C	Bek_E	Oikan	Kimon	Benten
pH	6.39 (−0.05)	5.95 (−0.06)	6.09 (−0.02)	5.67 (−0.09)	6.43 (−0.02)	6.36 (−0.03)	6.38 (−0.07)	6.29 (−0.05)
Cl^–^	285.21 (−66.15)	171.99 (−31.57)	390.12 (−33.14)	72.47 (−9.33)	107.42 (−3.44)	170.04 (−8.54)	79.73 (−2.25)	204.96 (−16.66)
NO_3_^–^	13.34 (−8.46)	0.08 (−0.08)	0 (0)	0.06 (−0.06)	0 (0)	0.58 (−0.2)	0.09 (−0.09)	8.77 (−3.33)
SO_4_^2–^	111.9 (−50.9)	14.99 (−3.2)	31.1 (−14.67)	3.14 (−0.78)	1.53 (−0.51)	3.87 (−0.79)	12.74 (−4.45)	72.38 (−12.85)
Na^+^	413.88 (−107.39)	251.12 (−31.13)	488.39 (−41.15)	142.16 (−10.97)	267.57 (−8.76)	284.49 (−12.11)	166.74 (−12.16)	386.86 (−28.16)
NH_4_^+^	26.58 (−4.81)	3.67 (−3.67)	2.8 (−1.48)	17.46 (−1.86)	16.89 (−4.17)	20.1 (−4.68)	40.26 (−2.65)	26.48 (−9.07)
K^+^	76.92 (−15.85)	46.24 (−11.64)	46.76 (−7.34)	33.44 (−7.65)	20.99 (−3.89)	42.06 (−5.56)	30.68 (−5.1)	44.9 (−13.81)
Mg^2+^	103.69 (−24.99)	49.98 (−2.8)	64.9 (−8.33)	30.96 (−2.4)	27.45 (−5.05)	43.77 (−3.35)	26.2 (−5.67)	38.17 (−5.5)
Ca^2+^	221.03 (−55.27)	57.7 (−4.65)	73.47 (−2.74)	64.5 (−8.12)	46.77 (−9.69)	74.36 (−10.5)	38.42 (−7.98)	70.98 (−4.16)
Si	223.33 (−56.56)	351.93 (−48.63)	215.87 (−66.78)	346.46 (−93.05)	300.44 (−57.11)	241.36 (−18.32)	349.37 (−20.17)	599.25 (−40.22)
DON	108.34 (−8.86)	178.62 (−20.74)	173.27 (−14.82)	169.16 (−18.52)	146.57 (−16.07)	140.39 (−7.83)	82.84 (−13.28)	75.25 (−3.2)
DTN	2.08 (−0.25)	2.55 (−0.25)	2.47 (−0.21)	2.61 (−0.26)	2.29 (−0.25)	2.26 (−0.11)	1.72 (−0.2)	1.55 (−0.14)
DTP	20.22 (−8.21)	98.33 (−20.91)	41.04 (−5.98)	32.26 (−6.05)	93.68 (−38.14)	22.97 (−4.36)	31.23 (−10.02)	14.19 (−1.82)

Ab.: Abbreviation of habitat name.

Means and SE are shown in the upper and under lines in parentheses, respectively.

The units of measurement for Cl^–^, NO_3_^–^, SO_4_^2–^, Na^+^, NH_4_^+^, K^+^, Mg^2+^, Ca^2+^, Si, and DON were μmol/L.

The units of measurement for DTN and DTP were μmg/L and μg/L, respectively.

**Table 2:** Summary of habitat characteristics based on concentrations of soil water chemistry using PCA.

	PC1	PC2	PC3	PC4	PC5	PC6	PC7
SD	2.44	1.97	1.07	1.05	0.84	0.36	0.27
PV	0.46	0.30	0.09	0.08	0.05	0.01	0.01
CP	0.46	0.76	0.84	0.93	0.98	0.99	1.00
Element							
pH	–0.18	–0.22	0.29	**–0.67**	–0.17	0.22	**–0.42**
Cl^–^	–0.26	0.29	**0.41**	0.01	0.29	–0.26	0.22
NO_3_^–^	**–0.38**	–0.08	–0.11	0.17	–0.28	**0.41**	0.00
SO_4_^2–^	**–0.39**	–0.01	–0.01	0.18	–0.20	0.01	**0.38**
Na^+^	–0.29	0.20	**0.53**	–0.01	0.14	0.26	0.13
NH_4_^+^	–0.11	**–0.44**	–0.30	–0.20	0.11	–0.16	**0.37**
K^+^	**–0.36**	0.18	–0.19	0.12	–0.06	**–0.48**	**–0.58**
Mg^2+^	**–0.34**	0.27	–0.15	–0.08	–0.08	–0.27	0.03
Ca^2+^	**–0.36**	0.13	**–0.35**	–0.07	–0.16	**0.31**	0.07
DTN	0.19	**0.43**	–0.24	0.02	–0.02	**0.31**	–0.01
DON	0.21	**0.43**	–0.06	0.05	–0.01	0.20	–0.12
DTP	0.23	0.18	0.19	–0.19	**–0.80**	–0.29	0.26
Si	0.01	**–0.33**	0.29	**0.63**	–0.26	0.06	–0.24

Standard deviation (SD), proportion of variance (PV), cumulative proportion (CP) of PCA, and rotated component matrix (elements).

Bold characters:| rotated component | > 0.30

### Relationship between leaf characteristics and habitat environmental conditions

Leaf mass per area (LMA) varied from 5.22 to 7.13 μg/cm^2^ (Table 3). After simplifying the Gaussian GLMs for the effect of sex and habitat environment on LMA among the seven populations, we found that LMA was positively affected by PC2 and negatively affected by PC3, but not affected by PC1 and sex (Table 4).

**Table 3:** Leaf characteristics.

	LMA	P/M	Porosity	δ^13^C
	(μg/cm^2^)	(mm/mm)	(%)	(‰)
Oike		0.466 (0.013)	55.5 (1.2)	-31.37 (0.35)
Po	6.35 (0.39)	0.558 (0.018)	81.4 (1.5)	-31.03 (0.09)
Ochiishi	7.13 (0.41)	0.558 (0.016)	78.7 (1.7)	-30.51 (0.22)
Bekanbeushi_center	7.13 (0.55)	0.537 (0.009)	72.0 (1.9)	-30.69 (0.20)
Bekanbeushi_edge	5.22 (0.27)	0.521 (0.197)	71.1 (2.8)	-30.70 (0.24)
Oikanamai	5.86 (0.64)	0.524 (0.018)	64.6 (2.2)	-32.14 (0.27)
Kimonto	5.40 (0.41)	0.559 (0.127)	71.9 (2.6)	-31.82 (0.33)
Bentennuma	6.87 (0.33)	0.543 (0.013)	79.8 (1.5)	-31.67 (0.51)

LMA: leaf mass per leaf area

P/M: thickness of the palisade layer per mesophyll layer

Porosity: mesophyll porosity

Values represent means with standard error in parentheses

**Table 4:** Standard partial regression coefficient for LMA and AIC based on the GLMs.

Explanatory variable	Full model	Best model
PC1	–0.236	
PC2	0.394	0.318
PC3	–0.510	–0.343
male vs. female	0.003	
AIC	6.9	4.6

**Fig 3:**
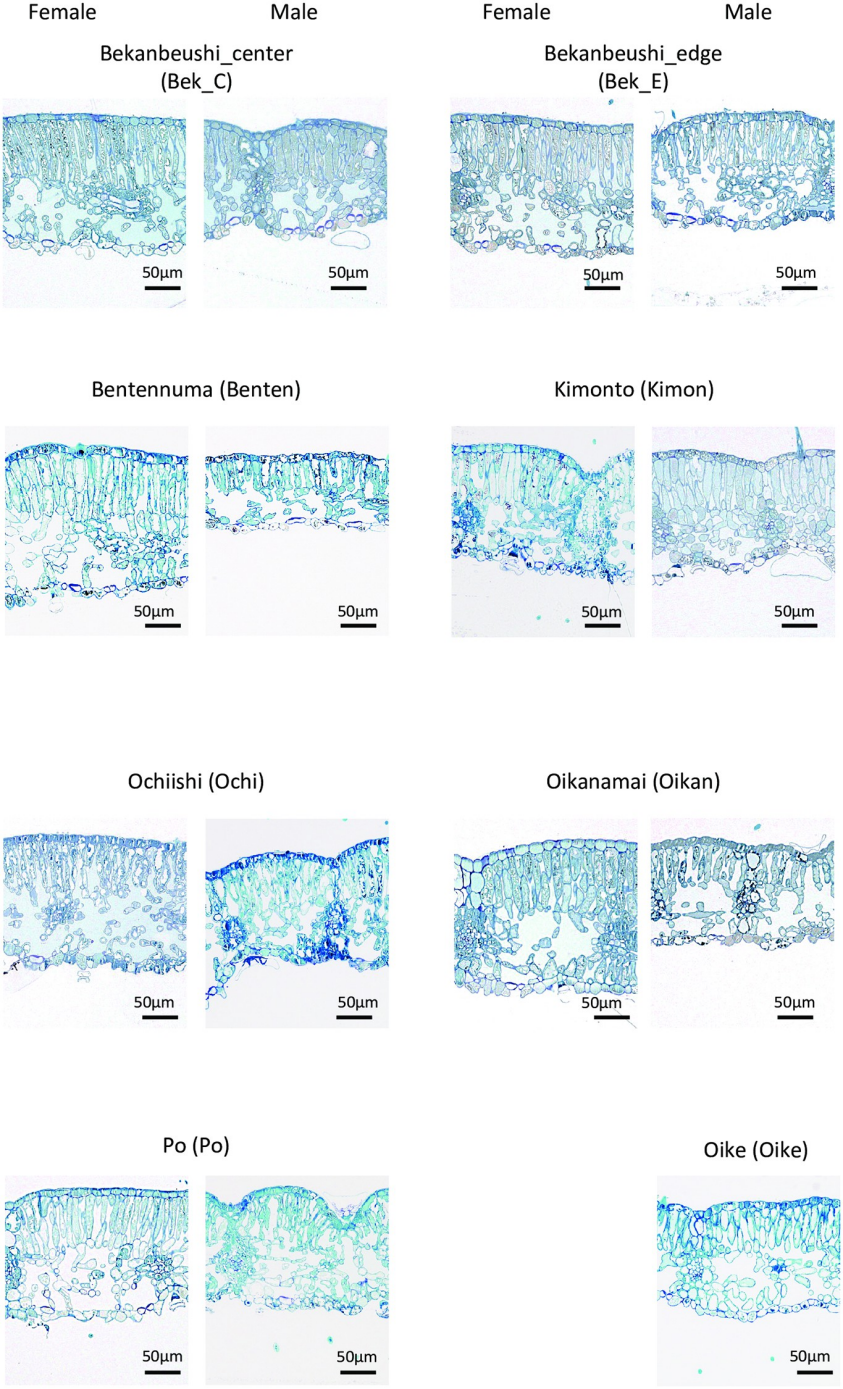
Transections of leaves of representative *Myrica gale*.

**Fig 4:**
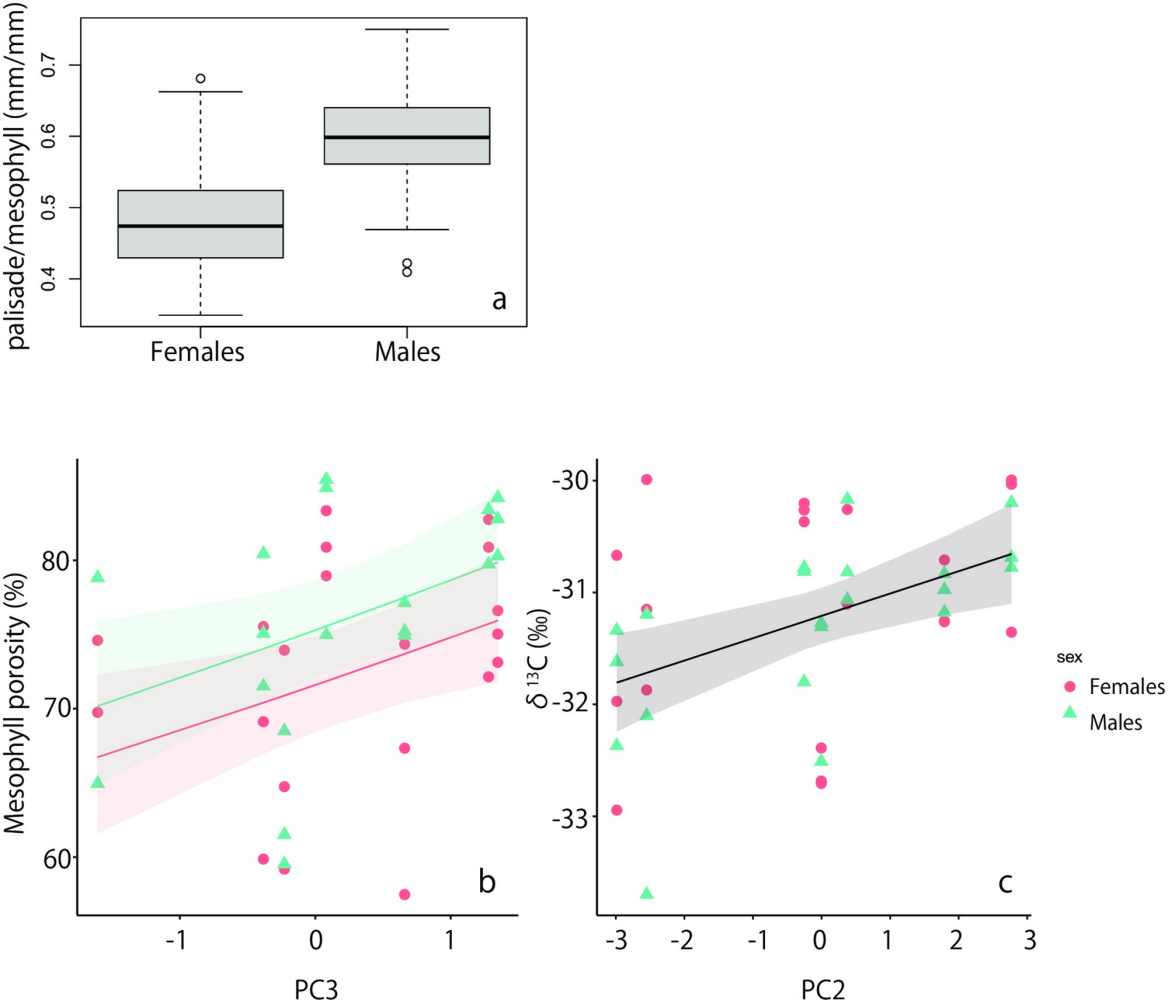
Relationship between thickness of palisade layer per mesophyll layer and sexes (a), mesophyll porosity, PC3, and sexes (b), and δ^13^C values and PC2 (c). The line and shading indicate the mean and the 95% confidence intervals of GLMs. Red circles and lines: females, blue triangles, lines: males.

The thickness of the palisade layer per mesophyll layer ranged from 0.466–0.559 mm/mm (Fig 3, Table 3). A Gaussian generalized linear mixed model was developed to determine the effects of sex and habitat environment (PC1–PC3) on the thickness of the palisade layer per mesophyll layer. Habitat environment did not affect these values. According to the lowest AIC model (AIC = −1349.3), the leaves of females had a thinner palisade layer per mesophyll layer than those of males (Fig 4a).

The mesophyll porosity values were over 50% for all eight populations (Table 3). There was no significant correlation between LMA and mesophyll porosity (test of no correlation, *p* = 0.170). After simplifying the log–linked Gaussian GLMs for the effect of sex and habitat environment on mesophyll porosity among the seven populations, we found that model considered the effects of PC3 and sex showed the lowest AIC (AIC = -89.4) among other models. Thus, mesophyll porosity was positively affected by PC3 and mesophyll porosity of males was larger than those of females (Fig 4b).

The δ^13^C values of leaves varied from −32.14 to −30.51 ‰ (Table 3). After simplifying the Gaussian GLMs for the effect of sex and habitat environments on δ^13^C values among the seven populations, we found that δ^13^C values were positively affected by PC2 but not by PC1, PC3, and sex (Fig 4c).

## Discussion

### Habitat environmental conditions

PC1 was negatively related to the concentrations of anions and cations in soil water. Concentrations of cations such as K^+^, Mg^2+^, and Ca^2+^ suggest that soil water was determined not only by precipitation but also by groundwater, because the source of high concentrations of cations is thought to be bed rock weathering substances in soil water [23]. Thus, PC1 was associated with the ionic components. PC2 was positively related to DON, which was calculated by subtracting NO_3_^−^ and NH_4_^+^ from DTN, and negatively related to NH_4_^+^; however, DON showed the opposite pattern for NH_4_^+^ in PC2. Inorganic N (NO_3_^−^ and NH_4_^+^) was very low in these habitats compared to that in other fens [24], and plants at such sites would utilize amino acids, which are one of the DON components in extremely N-limited sites [25]. One of the extremely N-limited sites, such as wetlands, provides ideal conditions for the removal of reactive nitrogen via denitrification [26]. Therefore, a lower PC2 was associated with poor N status sites and vice versa. PC3 revealed strong associations between Na^+^ and Cl^−^, indicating that PC3 is associated with sea salt contributions.

Po, Ochiishi, and Bekanbeushi_center were classified as high moors (bog), and Bekanbeushi_edge, Oikanamai, Kimonto, and Bentennuma were classified as low moors (fen) [27]. High moors, such as Po and Ochiishi, had high loadings on PC2, and lower moors, such as Kimonto and Bentennuma, had low loadings on PC2 (Fig 2). Our analysis suggests that high moors have rich N status. Oikanamai is an intermediate between high and low moors. Oike had low loadings on PC1 and was classified as a spring water minor [28]. Bekanbeushi_edge and Bekanbeushi_center had high loadings on PC1, suggesting that their water supply was mainly precipitation, although these areas were classified as different types of moors. In a previous study [16, 17], we focused on macronutrients, such as nitrogen, potassium, and phosphorous. However, the ratio of nutrients is sometimes more important for plants than the concentration itself [29, 30], and micronutrients deficiency sometimes affects leaf morphology and water use efficiency [31, 32]. We adopted a more realistic approach in field research using principal component analysis (PCA) and succeeded in distinguishing habitats using 13 elements.

### Relationship between leaf characteristics and habitat environmental conditions and sexes

**Fig 5:**
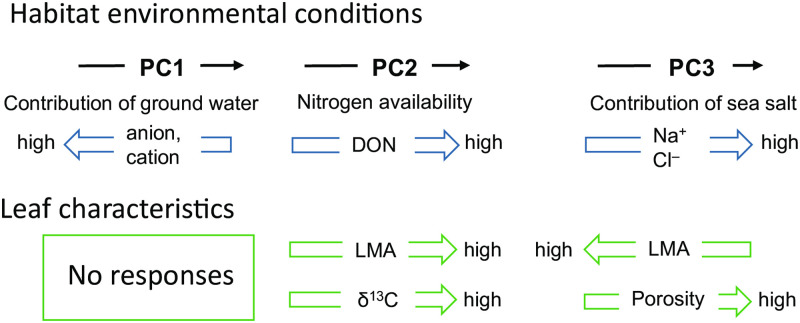
Schematic overview of the relationship between leaf characteristics and habitat environmental conditions.

Because the relationship between leaf characteristics and habitat environmental conditions in this study was complex, we have summarized in Fig 5. The LMA of a species is a good indicator of its leaf economic spectrum during resource acquisition [33]. The LMA of *M*. *gale* was positively related to PC2 and negatively related to PC3, but not to PC1 or sex. This suggests that the LMA decreased with poor N status and high sea salt contributions. LMA is the product of leaf density and leaf volume to area ratio [34, 35]. Under oligotrophic conditions, leaf density decreases, leading to a low LMA [36]. Thus, our results are consistent with those of the previous studies. Although *M*. *gale* coexisted with symbiotic N-fixing bacteria and there was no significant difference in N concentrations in leaves between sexes [17], the concentration of nitrogen in soil water positively affected the LMA. However, the habitat environment did not affect the thickness of the palisade layer per mesophyll layer, which is generally correlated with leaf density. In addition to the negative relationship between LMA and PC3, we found that mesophyll porosity increased in PC3. The physical structures in the leaves of *M*. *gale* in sites near the sea may be sparser. This is the first study to investigate the effects of salinity on the morphology and physiology of leaves in *M*. *gale*. Although the absolute concentrations of Na^+^ and Cl^−^ were not very high compared to those in other studies [37, 38], we need to clarify the effects of salinity stress on *M*. *gale* by conducting growth experiments under different salinity concentrations in the future.

### The effects of sex

We found that the leaves of females had a thinner palisade layer in the mesophyll layer than those of the males. In addition, mesophyll porosity was lower in females than in males. These sexual differences might affect the differences in photosynthetic ability between the sexes. Recently, anatomical differences in leaves between sexes were found under salt stress in the dioecious tree, *Populus cathayana* Rehder [39]. In salt-stressed females of *P*. *cathayana*, mesophyll spongy cell density increased, and the surface area of chloroplasts adjacent to the intercellular air space decreased. The results of sex differences in mesophyll porosity in our study were similar to those of the present study. In the future, we need to verify the differences between sexes in these characteristics and photosynthetic ability under certain environmental conditions.

### The effects of nitrogen and other nutrients

We found that the δ^13^C values of *M*. *gale* leaves were positively related to PC2, but not to PC1, PC3, or sex. This result indicated that the δ^13^C values of *M*. *gale* leaves became more negative under poor N conditions because PC2 was positively related to DON. Previous studies have reported that nutrient deficiency alters plant δ^13^C, where both an increase and decrease in δ^13^C are observed. For example, in tomatoes, the δ^13^C values of shoots increase with increasing P starvation [40]. Similarly, in barley, the δ^13^C values of shoots increase with decreasing Mg supply [32]. In contrast, in cotton, the δ^13^C values of shoots decrease with K deficiency [41]. The difference in δ^13^C alterations observed in these studies can be explained by the difference in the relative decrease in photosynthesis and stomatal conductance induced by nutrient deficiency. Leaf δ^13^C values reflect the long-term averaged ratio of photosynthesis (*A*) to stomatal conductance (*g*_s_), that is, long–term *A*/*g*_s_, wherein an increase in δ^13^C indicates an increase in *A*/*g*_s_, and vice versa [42]. When nutrient deficiency induced a more extreme decrease in *A* compared to *g*_s_, leaf *A*/*g*_s_ will decrease, and leaf δ^13^C will decrease. This may be the case in our study and in previous studies on Mg and P deficiency in barley and tomato, respectively [32, 40].

### Management of endangered population

In the absence of females, Oike had unique soil water chemistry among the study populations owing to the high concentrations of cations. Furthermore, only Oike had a high concentration of NO_3_^-^. Although Oike was well maintained under oligotrophic conditions until 2007 [16], eutrophication may have progressed because of vegetation succession of tall grass. However, leaf characteristics except for mesophyll porosity were not shown as there were several outliers. Although mesophyll porosity increased with PC3, the lowest values of mesophyll porosity at Oike might have different mechanisms from those of other populations because Na^+^ and Cl^−^ were not low. We need to monitor soil water chemistry and the growth and reproduction of the endangered Oike population.

## Conclusion

Our results confirm that sex and nutrient availability affected the leaf morphology and physiology of the dioecious shrub *M*. *gale*. The nitrogen availability in different habitats affected LMA, which decreased under poor nitrogen conditions. Nevertheless, *M*. *gale* coexisted with symbiotic N-fixing bacteria. Leaf morphology would change not only with nitrogen but also with salinity as sea salt contribution affected lower LMA and higher mesophyll porosity. On the other hand, soil water chemistry did not affect palisade thickness, and the leaves of females showed thinner palisade layers per mesophyll layer than those of males. Under poor nitrogen conditions, the δ^13^C of *M*. *gale* leaves decreased, suggesting that nutrient deficiency would further decrease under the long-term averaged ratio of photosynthesis to stomatal conductance and thus lead to low water use efficiency. In addition, further direct comparisons of the photosynthetic ability of *M*.*gale* between sexes and habitats are needed in the future.

We also found that *M*. *gale* grows in extremely oligotrophic environments and that reproductive habits, such as sex ratio at the flowering level, are suppressed by the phosphorus concentration in leaves and potassium concentration in soil water [16, 17]. This study suggests that nitrogen deficiency may affect the photosynthetic capacity and water use efficiency in both sexes. We showed that deficiencies of multiple nutrients have various effects at different growth stages.

## Supporting information

S1 Table Mean leaf mass per leaf area (LMA; μg / cm^2^), thickness of the palisade layer per mesophyll layer (P/M; mm/mm), and δ^13^C (‰) of male and female leaves in eight habitats of *Myrica gale*. (DOCX)
