# Supplementary material for: Effects of sex and soil water chemistry on leaf morphology and physiology of Myrica gale var. tomentosa
Source: PLoS One. 2022 Sep 22;17(9):e0275024. doi: 10.1371/journal.pone.0275024 (PMC9499279; doi:10.1371/journal.pone.0275024)
Supplement: S1 Table — (DOCX) [file pone.0275024.s001.docx]

S1Table. Mean leaf mass per leaf area (LMA; μg / cm^2^), thickness of the palisade layer per mesophyll layer (P/M; mm/mm), and δ^13^C (‰) of male and female leaves in eight habitats of *Myrica gale*.

| Habitat | sex | LMA | P/M | δ^13^C | Porosity |
| --- | --- | --- | --- | --- | --- |
| Oike | male | NA | 0.466 | -31.37 | 55.5 |
| Po | male | 5.98 | 0.623 | -30.99 | 81.8 |
| Ochiishi | male | 7.11 | 0.609 | -30.55 | 82.4 |
| Bekanbeushi_center | male | 8.06 | 0.558 | -30.68 | 72.9 |
| Bekanbeushi_edge | male | 5.06 | 0.617 | -31.13 | 75.8 |
| Oikananmai | male | 4.75 | 0.616 | -31.70 | 63.2 |
| Kimonto | male | 5.31 | 0.610 | -31.78 | 75.7 |
| Betennuma | male | 6.36 | 0.597 | -32.33 | 81.1 |
| Po | female | 6.72 | 0.494 | -31.07 | 81.1 |
| Ochiishi | female | 7.15 | 0.508 | -30.46 | 74.9 |
| Bekanbeushi_center | female | 6.21 | 0.516 | -30.68 | 71.0 |
| Bekanbeushi_edge | female | 5.38 | 0.426 | -30.28 | 66.4 |
| Oikananmai | female | 6.97 | 0.431 | -32.59 | 66.0 |
| Kimonto | female | 5.49 | 0.509 | -31.86 | 68.2 |
| Betennuma | female | 7.21 | 0.488 | -31.00 | 78.6 |
